# Orthodontic Treatment of a Mandibular Incisor Extraction Case with Invisalign

**DOI:** 10.1155/2014/657657

**Published:** 2014-06-12

**Authors:** Khalid H. Zawawi

**Affiliations:** Department of Orthodontics, Faculty of Dentistry, King Abdulaziz University, P.O. Box 80209, Jeddah 21589, Saudi Arabia

## Abstract

Mandibular incisor extraction for orthodontic treatment is considered an unusual treatment option because of the limited number of patients that meet the criteria for such treatment. Accurate diagnosis and treatment planning is essential to achieve the desired results. Adult orthodontic patients are increasingly motivated by esthetic considerations and reject the idea of conventional fixed appliances. In recent years, Invisalign appliances have gained tremendous attention for orthodontic treatment of adult patients to meet their esthetic demands. In this case report, a case of Class I malocclusion was treated with mandibular incisor extraction using the Invisalign appliance system. Successful tooth alignment of both arches was achieved. The use of Invisalign appliance is an effective treatment option in adult patients with Class I malocclusion that requires incisor extraction due to moderate to severe mandibular anterior crowding.

## 1. Introduction

Since its introduction in 1999, the Invisalign system (Align Technology, Santa Clara, CA, USA) has become an accepted treatment choice for clinicians because of comfort when compared with traditional orthodontic fixed appliances. Numerous reports have been published during the last decade, demonstrating the applicability of this system in correcting numerous types of malocclusions [1–6].

Even though the popularity of Invisalign is growing and it is being used in complex cases [2, 3, 7], questions still persist regarding the appropriate use of this system and its limitations. Various disadvantages and limitations have been outlined, owing to the characteristics of the material used and the thermoforming process, which in certain cases may constrain or even render using these clear aligners very difficult [7, 8].

Mandibular incisor extraction in orthodontic treatment is unusual. Even though this option was abandoned for years for more conservative approaches, it is necessary sometimes in dental crowding to restore dental arch symmetry by sacrificing teeth. This procedure constitutes a favorable alternative in treating certain clinical situations where the therapeutic aims need to be adjusted. Hahn in 1942 [9] advocated the removal of mandibular incisor to reduce the number of anterior teeth. Albeit it is not the standard approach, extraction of mandibular incisors constitutes a therapeutic alternative when treating certain orthodontic cases. This procedure allows orthodontists to improve occlusion and dental esthetics with minimal orthodontics [10].

There is a scarcity in the literature regarding single mandibular incisor extraction, perhaps due to the limited number of patients who meet the standards for such management. There are diagnostic criteria that are usually required for single mandibular incisor extractions: (1) Class I molar relationship, (2) moderate crowding in the mandibular incisors, (3) mild or no crowding in the maxillary anterior teeth, (4) acceptable soft-tissue profile, (5) minimal to moderate overjet and overbite, (6) minimal growth potential, and (7) a tooth-size discrepancy, for example, peg-shaped or missing maxillary lateral incisors [11].

However, before embarking on this procedure, a full diagnostic set-up is essential to predetermine an accurate outcome and confirm that the occlusal results will be adequate. Unfortunately, these diagnostic set-ups involve frequently extensive and strenuous laboratory procedures when cutting teeth from the models and setting and waxing the teeth in alignment. Moreover, conventional techniques of tooth repositioning using removable appliances involve alteration of the casts by resetting each tooth or by scraping the plaster away from the teeth to be moved and blocking out space with wax [11, 12].

Therefore, this case report was aimed at illustrating the use of the Invisalign system in the treatment of an adult patient with severe mandibular anterior crowding after incisor extraction.

## 2. Case Report

A 56-year-old male presented with a chief complaint of “my lower teeth are crocked.” His dental history included multiple restorations, fixed prosthetic, and root canal treatments.

Clinical examination revealed complete lip competency with small chin and no mentalis muscle strain. His profile was convex and on smiling, he displayed almost 80% of his maxillary incisors with no gingival display. Molars and canines were in Class I relationship. The overbite was 60% and overjet was 4 mm, with the maxillary and mandibular midline coincident to one another and to the face. Oral hygiene was fair although there was localized mild gingival recession. The maxillary arch was well aligned, with mild anterior crowding. Bolton analysis indicated a maxillary dental excess of 1 mm. There was 6 mm of crowding in the mandibular anterior region.

**Figure 1:**
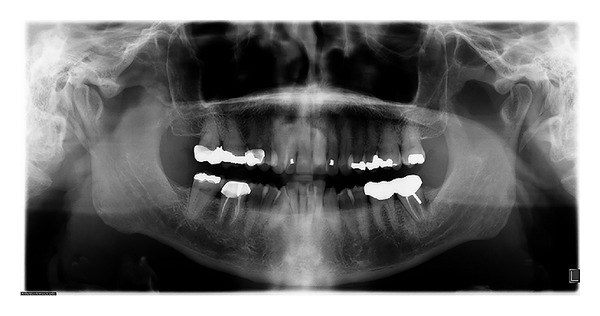
Initial panoramic radiograph.

The panoramic radiograph showed a full permanent adult dentition with multiple restoration and root canal treatments. There was mild generalized bone loss and root morphology was within normal limits (Figure 1). Cephalometric findings included a well-positioned maxilla and mild retrognathic mandible, resulting in mild Class II skeletal pattern. The maxillary and mandibular incisors were proclined and protruded.

### 2.1. Treatment Planning

The primary objective of the treatment was to address the patient's chief complaint, that is, to resolve the mandibular crowding and improve the overjet while avoiding more proclination of the mandibular incisors.

Several treatment alternatives were presented to the patient. The first was to extract all first premolars in both arches to alleviate the crowding and to achieve proper inclination and position of the anterior teeth. The problem with this option was that it was too aggressive.

The second option was to alleviate the maxillary and mandibular crowding by interproximal reduction using the air rotor stripping technique [13, 14]. However, the mandibular anterior teeth were not suitable for interproximal reduction due to their small size and shape. Furthermore, air rotor stripping of the posterior segment was not a suitable choice because of the Class I occlusion and presence of posterior crowns.

The third option was to extract a mandibular incisor to relieve the crowding. This plan would maintain the mandibular incisors in their current position and inclination while maintaining the Class I molar and canine relationship. A diagnostic wax set-up was made to confirm this treatment option. The major drawbacks to this option were that a mandibular incisor would have to be extracted, the mandibular midline would be lost, and the overjet could be increased.

The patient was presented with all options and he agreed to the mandibular incisor extraction using the Invisalign appliance. The mandibular left central incisor was selected in this case because it was the most malaligned and lingually positioned and thus contributed most to the crowding, and also the attached gingiva was the least healthy of all the mandibular incisors.

### 2.2. Treatment Progress

**Figure 2:**
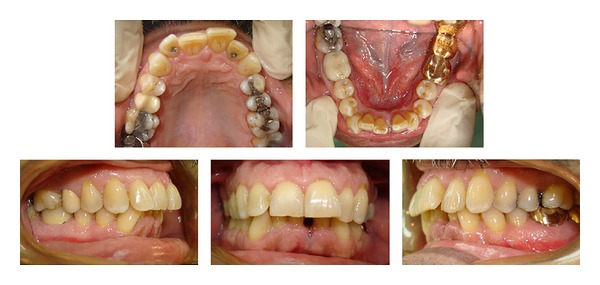
Intraoral composite photographs following the extraction of the mandibular left central incisor.

After the mandibular left central incisor was extracted (Figure 2), maxillary and mandibular polyvinyl siloxane impressions were taken and sent to Invisalign to fabricate the aligners. A proprietary Align Technology software ClinCheck system (Align Technology, Santa Clara, CA, USA) generated the patient's tooth set-up and stages of tooth movements in three dimensions and was reviewed by the orthodontist on a computer.

**Figure 3:**
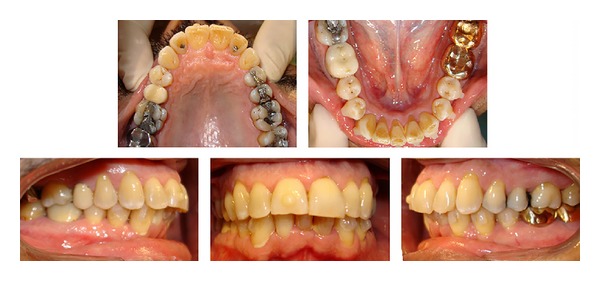
Progress intraoral composite photographs showing the bonded attachments.

Before the delivery of the first aligner, vertical composite attachments were bonded to the mandibular teeth to prevent tipping during space closure and a button attachment was bonded on the maxillary right central (Figure 3). The patient was seen every four weeks for delivery of new aligners and to monitor treatment progress and aligner fit. The patient was instructed to change to the next set of aligners every 2 weeks. Twelve aligners were required in the maxillary arch and 20 for the mandibular. Interproximal reduction was required in the maxillary lateral and central teeth with a total of 0.5 mm of reduction on each side.

Total treatment time was 10 months. The patient was then given Essex-type maxillary and mandibular retainers to be worn at night.

### 2.3. Treatment Results

**Figure 4:**
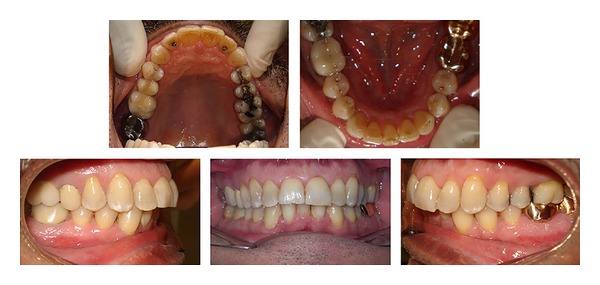
Posttreatment intraoral photographs.

The Class I molar and canine relationships were maintained. The mandibular extraction space was completely closed and the maxillary incisors were well aligned. Even though interproximal reduction was performed between the maxillary incisors, the overjet was not changed and was acceptable to the patient. The gingival recession in the mandibular right central incisor region did not change during treatment and both arches showed good alignment (Figure 4).

**Figure 5:**
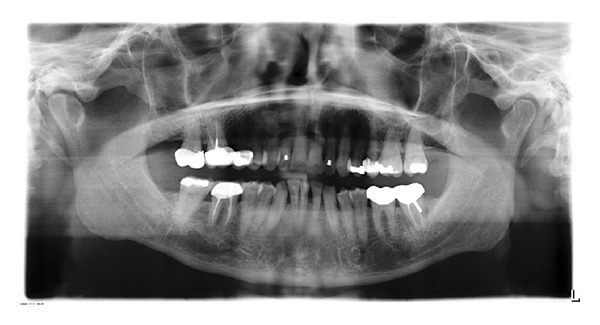
Posttreatment panoramic radiograph.

The posttreatment panoramic X-ray revealed a relatively well-aligned upper and lower incisor roots (Figure 5).

## 3. Discussion

The main treatment goals were achieved and the patient's chief complaint was addressed. The molars and canines were in Class I occlusion, and the mandibular anterior dental crowding, the patient's main complaint, was corrected.

Mandibular incisor extraction poses important limitations that should be taken into consideration. An increased overjet is a contraindication to this procedure. Moreover, the mandibular canine may displace mesially leading to loss of canine function protection.

Invisalign offered an invisible and comfortable treatment option for closing the mandibular extraction spaces and alignment of the maxillary incisors. Simulated space closure of the mandibular incisors in the ClinCheck analysis was of great aid during treatment planning. However, it should be stressed that continuous monitoring during the retention phase is necessary to prevent space reopening [5]. The retention protocol after treatment with Invisalign appliances may be similar to that in cases treated with fixed appliances [5] or Vivera retainer.

The decision of which incisor to extract is always important. Several considerations should be assessed, including the presence of gingival recession or periodontal defect, large restoration, the location of incisor relative to the crowding, and the mesiodistal width of the incisor. Usually the lateral incisor is the preferred tooth; however, the incisor that is outside the natural arch and closest to the crowding is frequently the candidate for extraction [12]. Mandibular incisor extraction could also be considered when the individual has congenitally missing maxillary lateral incisors along with moderate to severe mandibular anterior crowding [11].

The Invisalign system requires taking impressions with polyvinyl siloxane impression material for longer shelf life, superior accuracy, and multiple model pours. Full-arch impressions can occasionally be challenging to take with this material; however they are essential to this technique. Invisalign treatment requires the clinician to plan the sequential tooth movements from start to end, which is a rather different process than with conventional fixed appliances. The ClinCheck software allows careful and critical evaluation of the entire treatment by the clinician in all three dimensions. In the current case report, mandibular incisor extraction was the appropriate choice. The active treatment time was almost equivalent to that of fixed appliance therapy and therefore offers support of a viable alternative to conventional techniques.

## 4. Conclusion

The use of Invisalign appliance is an effective and esthetic treatment option in adult patients with Class I malocclusion that requires incisor extraction due to moderate to severe mandibular anterior crowding.
